# Supplementary material for: The Neuropeptide Cortistatin Alleviates Neuropathic Pain in Experimental Models of Peripheral Nerve Injury
Source: Pharmaceutics. 2021 Jun 24;13(7):947. doi: 10.3390/pharmaceutics13070947 (PMC8309056; doi:10.3390/pharmaceutics13070947)
Supplement: Supplementary file 1 [file pharmaceutics-13-00947-s001.zip › pharmaceutics-1241480-supplementary.pdf]

pharmaceutics

MDPI

Supplementary Materials: The Neuropeptide Cortistatin Alleviates Neuropathic Pain in Experimental Models of Peripheral Nerve Injury

Clara P. Falo, Raquel Benitez, Marta Caro, Maria Morell, Irene Forte-Lago, Pedro Hernandez-Cortes, Clara Sanchez-Gonzalez, Francisco O'Valle, Mario Delgado and Elena Gonzalez-Rey

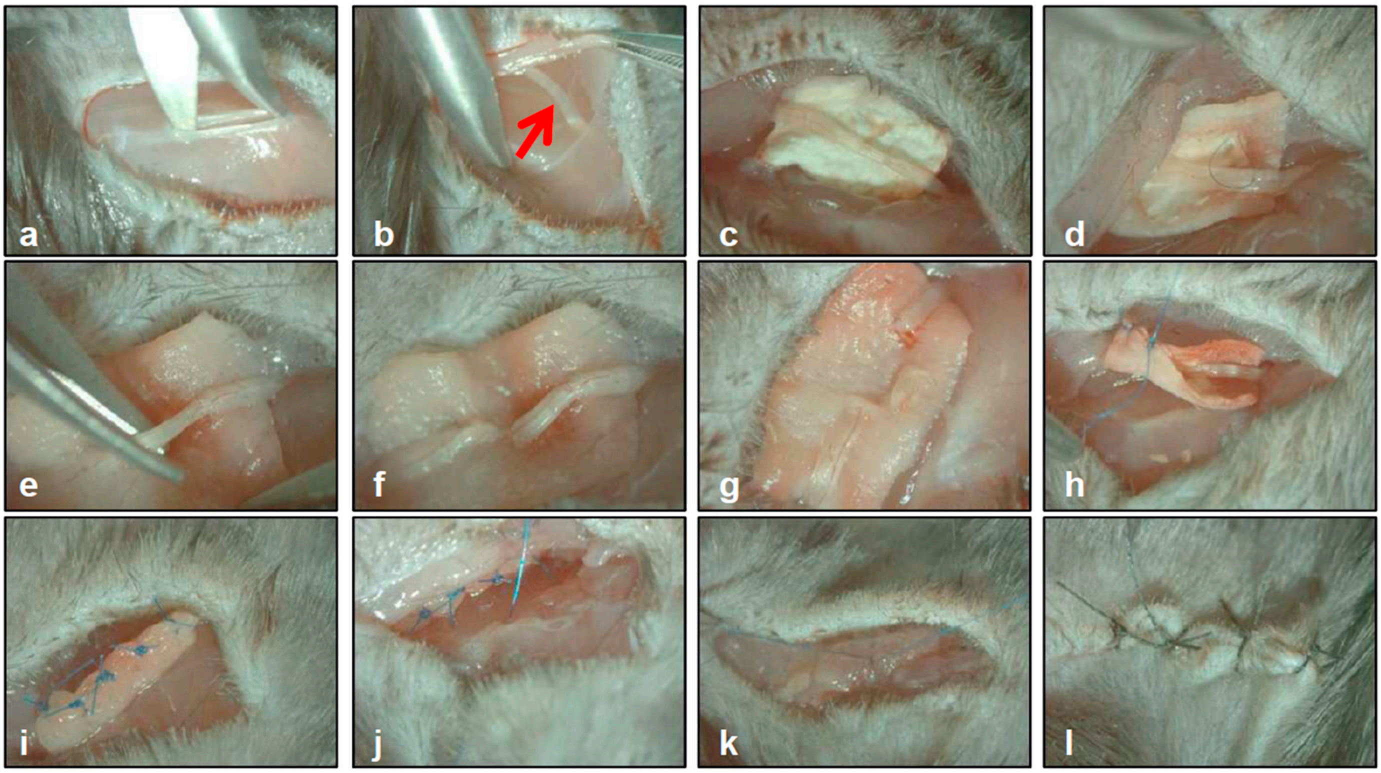

Figure S1. Intraoperative photographs of surgical transection and tubulization of the sciatic nerve: (**a**) mice suffered an incision in the skin and (**b**) sciatic nerve was exposed; (**c**–**d**) a semipermeable resorbable collagen membrane (4x4 mm) was collocated under the nerve and was sutured into the distal and proximal nerve ends before sectioning; (**e**–**g**) the sciatic nerve was sectioned at 1mm from the sutures and 2 mm segment of the distal portion of the nerve was removed; (**h**–**i**) the membrane wrapped the transected nerve and it was closed forming a tubular conduit; (**j**–**k**) the muscular layer was closed around the tube; (**l**) skin was sutured for closing the surgical site.
